# The double-edged scalpel: Experiences and perceptions of pregnancy and parenthood during Canadian surgical residency training

**DOI:** 10.1371/journal.pone.0301190

**Published:** 2024-03-27

**Authors:** Mikaela J. Peters, Alissa W. Zhang, Darren M. Roffey, Kelly A. Lefaivre

**Affiliations:** 1 Department of Orthopaedics, Faculty of Medicine, University of British Columbia, Vancouver, British Columbia, Canada; 2 MD Undergraduate Program, Faculty of Medicine, University of British Columbia, Vancouver, British Columbia, Canada; 3 Division of Orthopaedic Trauma, Vancouver General Hospital, Vancouver Coastal Health, Vancouver, British Columbia, Canada; Kasturba Medical College Mangalore, Manipal Academy of Higher Education, INDIA

## Abstract

**Introduction:**

Only 34% of Canadian surgeons in 2022 were female. The protracted length of surgical residency training, concerns regarding infertility, and increased rates of obstetrical complications have been shown to contribute to the disproportionate lack of females in surgical specialties.

**Methods:**

A novel online survey was sent to all surgical residents in Canada. Respondents were asked about perceptions of pregnancy and parenthood during surgical training, and parents were asked about parental leave, accommodations they received, and pregnancy complications. Chi squared tests were used to compare opinions of male and female residents.

**Results:**

A total of 272/2,419 (11.2%) responses were obtained, with a high response from females (61.8%) and orthopaedic residents (29.0%). There were 56 women reporting 76 pregnancy events during training, 62.5% of which had complications. Notably, 27.3% of men and 86.7% of women ‘agreed’ or ‘strongly agreed’ that surgeons have higher pregnancy complication rates than the general population (p<0.001). Men were much less likely to believe that pregnant residents should be offered modified duties (74.2% of men, 90.0% of women, p = 0.003). Women were much more likely to experience significant stigma or bias due to their status as a parent (43% of women, 0% of men, p<0.001). Women reported negative comments from others at a higher rate (58.5% of women, 40.7% of men, p = 0.013). Women believe there is negative stigma attached to being pregnant during training (62.7% of women, 42.7% of men, p = 0.01). The limitations of our study include a small sample size and response bias.

**Conclusion:**

Challenges and negative perceptions exist around pregnancy and parenthood in surgical residency, which disproportionately affect women trainees.

## Introduction

Although the majority of medical students in Canada are now women, only 34% of Canadian attending staff surgeons in 2022 were female [1]. Encouragingly, Canadian surgical residency programs are getting closer to achieving gender equity: in 2022–23, just over half of Canadian surgical residents were female [2]. Alas, there remains significant heterogeneity between surgical subspecialties; in particular, orthopaedic surgery and urology lag far behind, with just under one third of residents identifying as female [2].

Surgical trainees experience significant barriers during their training with respect to pregnancy and parenthood, and are at significantly increased risk of infertility and pregnancy complications compared to the general population [3, 4]. Surveys of program directors as well as orthopaedic surgery residents in the United States highlight negative perceptions amongst both male and female residents and staff regarding pregnancy and parenthood [5, 6]. Similarly, studies have shown that concerns regarding work-life balance and family life are a significant deterrent for women considering a career in surgery [7]. Increased length of surgical residency training compared to non-surgical specialties, infertility associated with delaying pregnancy, and increased rates of obstetrical complications in female surgical residents contribute to the disproportionate lack of females in surgical subspecialties [4, 8–10].

There has been a surge in literature investigating gender disparity in surgery over the last five years [11–15]. Many of the studies examining parenting perceptions and experiences are conducted in the United States, which has a vastly different work culture and parental leave policies than Canada. Fortunately, there is new research being published that focuses on the culture in Canada. Recent studies have shown Canadian female otolaryngologists-head and neck surgeons and Canadian plastic surgeons face challenges in family planning, ability to conceive, and breastfeeding [16, 17], while a scoping review showed that both female residents and staff surgeons experience significant and unique barriers before, during, and after motherhood that impact their personal and professional lives [18]. In order to continue to attract and retain qualified post-medical graduates and promote gender equality in the surgical specialties, it is imperative to create a supportive culture surrounding pregnancy and parenthood during surgical training.

The aim of our study was to utilize a cross-sectional survey to gather insights, perceptions and experiences of parenthood during surgical training from current trainees at accredited Canadian surgical programs. We hoped to provide important insight into the challenges faced by surgical resident parents, and to inform necessary cultural and policy changes to improve supports available.

## Methods

### Study design

The study protocol was conducted in accordance with the Strengthening the Reporting of Observational Studies in Epidemiology (STROBE) guidelines. Ethics approval was obtained from The University of British Columbia (UBC) Behavioural Research Ethics Board (H22-01963). As outlined in the Letter of Initial Contact and then again in the study Consent Form, informed consent was obtained in the context of administering the survey. If the respondent clicked on the link to open the survey, proceeded to answer the questions, and pressed “SUBMIT” to upload their responses, then their informed consent was considered to have been provided. Participants who clicked on the link were told they could choose to pull out of the study prior to submission by closing their internet browser, but they were not able to withdraw after their survey responses had been submitted.

The survey was distributed across all surgical training programs at accredited Canadian University training institutions in the 2022–2023 academic year. Surgical programs, as previously defined, included: cardiac surgery, general surgery, neurosurgery, obstetrics and gynaecology, orthopaedic surgery, otolaryngology, plastic surgery, urology, and vascular surgery [11]. Residents were invited to participate voluntarily in the online survey via email after receiving an invitation from their local program administrator. In order to increase our response rate, the study was promoted on institutional social media accounts and snowball sampling was used to extend the invitation to participate to current surgical fellows. Study data were collected anonymously and voluntarily from participants between August 10, 2022, to November 2, 2022 using the UBC Survey Tool provided by Qualtrics (Qualtrics, Provo, Utah, USA) [19].

### Outcome measures

A novel online survey was developed by the research team. We used a combination of qualitative interviews with other orthopaedic surgeons to ensure question clarity and a review of the existing literature, in an iterative modified-Delphi process, to develop our survey questions. We gathered demographic information on all participants (program type, postgraduate year (PGY), gender identification) and administered a series of questions exploring perceptions of pregnancy and parenthood in the context of surgical training to the entire study group. We had sections of the survey specific to residents who had a child during training regarding their experiences of pregnancy, parenthood, and parental leave during their training. Residents who had been pregnant during training were also asked about their pregnancy complications. The survey format was comprised of open-ended answers, Likert scale ratings, yes/no questions, and multiple-choice answers. The survey can be found in S1 File.

### Data preparation and statistical analysis

Continuous data were summarized with mean and standard deviation (SD). Likert scale answers were batched into three categories: agree (agree or strongly agree), neutral, and disagree (disagree or strongly disagree). Categorical data were summarized using frequency and percentages. Outcomes were compared between male vs. female participants, and parents vs. non-parents using Wilcoxon rank sum tests for continuous data and Chi-square or Fisher’s exact tests for categorical data. Analyses were conducted in R version 4.3.0 [20].

## Results

### Demographics

A total of 272 responses were obtained out of a total of 2,419 surgical residents in Canada in 2022 [2], representing a participation rate of 11%. There were 168 residents who identified as female (61.8%). A higher response rate was received from orthopaedic (29.0%), obstetrics and gynaecology (24.3%), and general surgery residents (21.7%). The majority of respondents had no children (67.5%). Responder demographics are summarized in Table 1. There were no statistically significant differences between the proportion of male and female respondents who had children.

**Table 1:** Responder demographics.

Demographic	Overall	Male	Female
**Respondents, n**	272	103	168
**Age, mean (SD)**	30.30 (3.39)	29.60 (2.46)	30.68 (3.76)
**Surgical specialty, n (%)**			
Cardiac surgery	7 (2.6)	6 (5.8)	1 (0.6)
General surgery	59 (21.7)	19 (18.4)	40 (23.8)
Neurosurgery	4 (1.5)	3 (2.9)	1 (0.6)
Obstetrics and gynecology	66 (24.3)	5 (4.9)	61 (36.3)
Orthopaedic surgery	79 (29.0)	41 (39.8)	38 (22.6)
Otolaryngology	15 (5.5)	6 (5.8)	9 (5.4)
Plastic surgery	14 (5.1)	5 (4.9)	9 (5.4)
Urology	19 (7.0)	15 (14.6)	4 (2.4)
Vascular surgery	9 (3.3)	3 (2.9)	5 (3.0)
**Children during training, n (%)**			
0	183 (67.5)	67 (65.0)	116 (69.0)
1	49 (18.1)	20 (19.4)	29 (17.3)
2	34 (12.5)	12 (11.7)	22 (13.1)
3	2 (0.7)	1 (1.0)	1 (0.6)
4+	3 (1.1)	3 (2.9)	0 (0.0)

**Fig 1:**
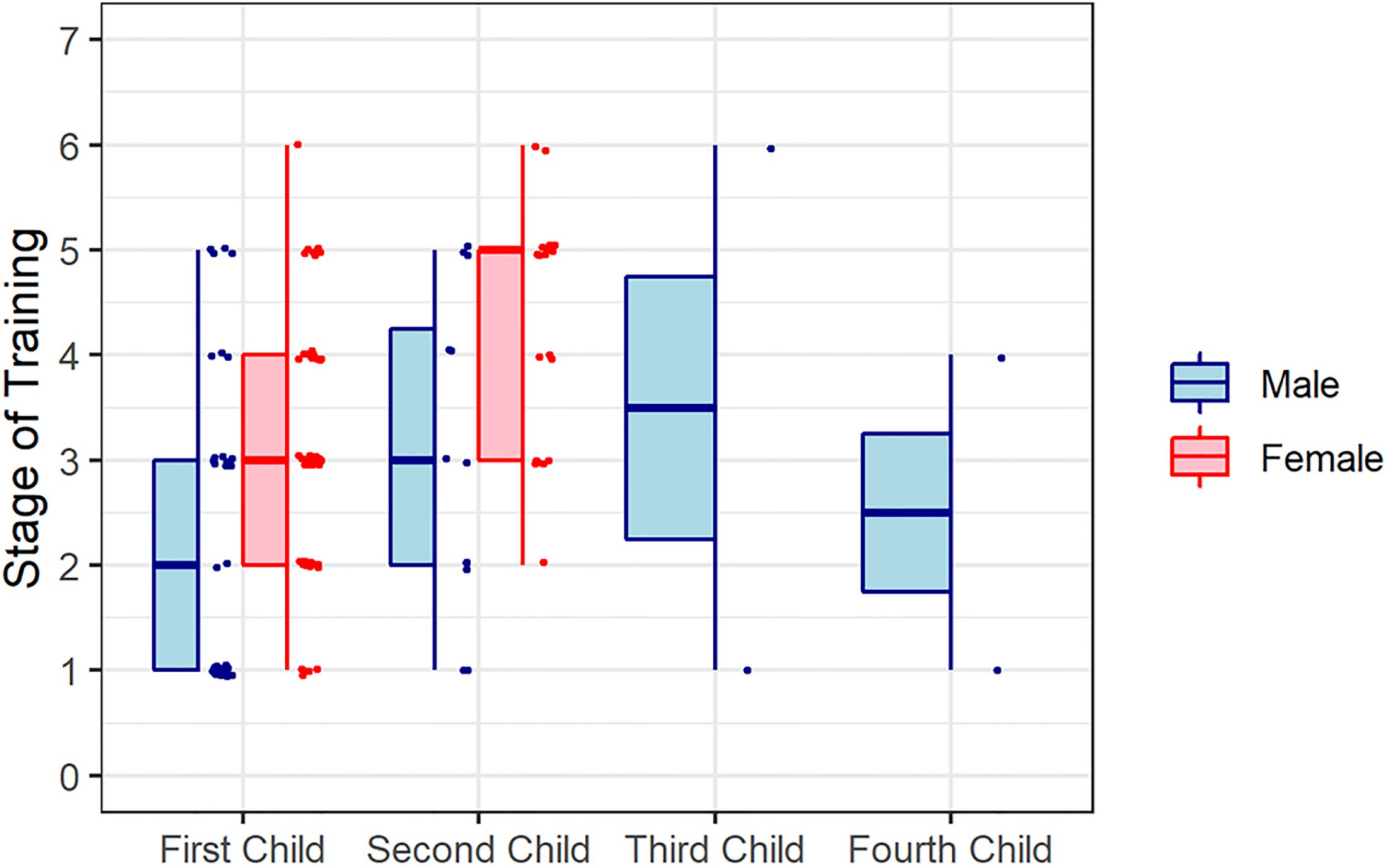
Timing of children.

Male residents started having children earlier than female residents, with their first child being born at a mean of PGY 2.34 (SD 1.43), while women’s first children were born at PGY 3.08 (SD 1.23, p = 0.01). Timing of children is summarized in Fig 1.

### Pregnancy outcomes

Out of the 56 reported pregnancies, 27 had at least one complication (62.5%); the most common was low birth weight (14.5% of pregnancies). Male residents were less likely to know about increased pregnancy complications in surgeons: only 27.3% agreed with the statement that surgeons have higher pregnancy complications than the general population, compared with 73.3% of female residents who agreed (p<0.001). Despite this, three quarters of males and 90% of females agreed that pregnant residents should be offered accommodations recommended by their care team, including reduction or cessation of call, and reduced standing time. Pregnancy outcomes are summarized in Table 2.

**Table 2:** Pregnancy perinatal complications.

**Number of pregnant women**	56
**Number of Complications, n (%)**	
0	21 (37.5)
1	18 (32.1)
2	8 (14.3)
3	2 (3.6)
4+	7 (12.5)
**Number of pregnancy events**	76
**Individual complications, n (%)**	
Gestational diabetes	6 (7.89)
Intrauterine growth restriction	7 (9.21)
Pre-eclampsia	6 (7.89)
Premature labour	5 (6.58)
Infections (UTI, GBS, influenza, COVID 19)	4 (5.26)
Stillbirth or neonatal loss	2 (2.63)
Low birth weight	9 (11.84)
Placenta previa	2 (2.63)
Postpartum anxiety or depression	7 (9.21)
Pregnancy induced hypertension	5 (6.58)
Other	20 (26.32)

### Decisions about parenthood

One third (33/97) of men and 44% (72/162) of women reported delaying becoming a parent due to their residency training. The most common reason in both genders was concern about lack of quality time to spend with family, followed by a desire to avoid missing training time. Men were more likely to report concern about lack of time to spend with family than women, while 41% (29/70) of female residents reported delaying parenthood due to concern about negative perception by faculty and/or residents, compared with 28% (9/32) of male residents. Only one fifth of male residents and one quarter of female residents were satisfied or very satisfied with their decision to delay becoming a parent, with no statistically significant difference between genders. Almost a third of residents (55/184) would have had a child or more children if they felt they had more support from their program or program director, with no statistically significant difference between genders. Nearly all responders believed that residents should be offered maternity or paternity leave, but the majority of residents also believed that reduced work hours during pregnancy and maternity leave negatively impact the workload of co-residents. Perceptions of parenthood in surgical training are summarized in Table 3. Data regarding parenthood experiences is summarized in Tables 4 and 5.

**Table 3:** Perceptions of parenthood in surgical training.

Statement	Male; agree or strongly agree, % (n)	Female; agree or strongly agree, % (n)	P-value
**Reduced work hours during pregnancy and maternity leave negatively impact the workload of co-residents**	61.8% (55)	58.3% (88)	0.10
**There is a negative stigma attached to being pregnant during training**	42.7% (38)	62.9% (95)	0.01
**Pregnant residents should be offered modified duties including reduction/cessation of call, reduced standing, and other accommodations recommended by their care team**	74.2% (66)	90.1% (136)	0.003
**Trainees should make up any call they miss during pregnancy or parental leave**	16.9% (15)	3.3% (5)	<0.001
**Residents should be offered maternity leave**	93.3% (83)	100% (151)	0.005
**Residents should be offered paternity leave**	93.3% (83)	100% (151)	0.005
**Surgeons exhibit an increased rate of complications during pregnancy compared to the general population**	27.3% (24)	73.5% (111)	<0.001
**Parental leave during training affects competency at program completion**	25.8% (23)	8.6% (13)	<0.001
**Parenthood during training affects competency at program completion**	19.1% (17)	8.6% (13)	0.003
**Pregnancy during training has an impact on future job prospects**	21.3% (17)	31.8% (48)	0.05
**Parenthood during training has an impact on future job prospects**	21.3% (19)	29.8% (45)	0.15

**Table 4:** Parental leave and modified work hours.

Description	Male	Female	P-value
**Length of leave (Weeks), mean (SD)**	2.9 (6.5)	34.8 (15.3)	<0.001
**Satisfaction with length of leave (satisfied or very satisfied); % (n)**	34% (14)	68.8% (44)	0.010
**Reduced hours after returning to work or birth of child; % (n) childbirth events**			0.27
Yes	0.0% (0)	9.0% (6)	
No	10.3% (3)	77.6% (52)	
Somewhat	89.7% (26)	13.4% (9)	
**Experienced stigma or bias due to status as a parent; % (n) parents**			<0.001
Yes	0.0% (0)	43.5%(20)	
Maybe	19.4% (6)	13.0% (6)	
No	81.6% (25)	43.5% (20)	

**Table 5:** Delaying parenthood.

Question	Male	Female	P-value
**Delayed becoming a parent due to residency; % (n)**	34.0% (33)	44.4% (72)	0.128
**Would have had a child or more children if you had more support from the residency program/residency program director; % (n)**	31.0% (22)	29.5% (33)	0.957
**Heard negative comments related to pregnancy during training from staff or co-residents; % (n)**	40.7% (35)	58.5% (86)	0.013
**What was the reason for delaying becoming a parent; % (n)**			
Personal preference	37.5% (12)	28.6% (20)	0.502
Wanted to avoid missing training time	68.8% (22)	68.6% (48)	1.000
Concern about lack of quality time to spend with family	90.6% (29)	70.0% (49)	0.043
To avoid negative perception by faculty and/or co-residents	28.1% (9)	41.4% (29)	0.285
**How satisfied were you with the decision to delay becoming a parent (satisfied or very satisfied); n (%)**	35.5% (11)	51.4% (36)	0.05

### Experiences of parents

Men took a median of 2 weeks of parental leave (range 0–2 weeks), while women took a median of 34.7 weeks of leave (range 24–52 weeks). Women were overall satisfied with the length of their leave, while men were less satisfied (p = 0.01). Women were much more likely to report stigma or bias due to their status as a parent; 43.5% of females reported certainly experiencing stigma or bias while no males reported stigma or bias. Women were more likely to report hearing negative comments related to pregnancy from staff or co-residents, with 58.5% of females reporting such comments compared to 40.7% of males (p = 0.013). Women were more likely to believe that there is a negative stigma associated with pregnancy during training, with 62.9% of women and 42.7% of men agreeing or strongly agreeing with this statement (p = 0.01). Men without children were significantly more likely to have a stay-at-home partner (p<0.001).

## Discussion

There is significant overlap between peak childbearing age and the chronological years spent in surgical training. In 2021, Canadian data showed that the mean age of a surgical resident in their final year of training was 33.3 years, while the mean age of mothers at time of delivery was 31.4 years [2, 21]. It is well-known that there are significant challenges combining parenthood and surgical training. A recent perspective piece in the New England Journal of Medicine calls for the importance of “acknowledging the biologic realities of pregnancy and committing to gender equity” [22].

Surgical trainees face unique challenges when considering starting a family in residency. A study of American plastic surgeons showed that women were twice as likely as men to delay childbearing due to training, at rates of 72% and 39% respectively [23]. In our study, over 40% of residents chose to delay becoming a parent due to residency, and less than a quarter of them were satisfied with this decision, with no significant difference between genders. By comparison, a recent study of Canadian plastic surgeons indicated most often choose to have children after their training is complete and choose to take shorter parental leaves as their careers progress [17].

Both male and female residents were concerned about missing training time, while men were more concerned about lack of quality time to spend with family. The fact that male residents took a median of 2 weeks of parental leave while female residents took a median of 34.7 weeks is in keeping with parental leaves in Canada. Statistics Canada indicated that in 2021, 48% of Canadian fathers took 5 weeks or less of paternity and/or parental leave, while 83% of Canadian mothers 27 to 52 weeks of maternity and/or parental leave [24]. While financial concerns may have affected the decision to take parental leave, it is difficult to postulate the reason(s) behind the stark difference between male and female residents; that trainees were concerned about avoiding negative perception by faculty and/or co-residents could be a factor. Notably, surgical residents provided the following commentary: “When discussing having children in residency with staff I have been directly told it would make me a worse resident/surgeon if I have them in residency, and I should delay.”; and “I feel pressured to have kids since I will be 35 when I finish residency, but training is so toxic and we have so little control over our lives that I just want to get it over with before trying to have kids.”

Although both women and men face challenges in starting a family during training, these challenges disproportionally affect women. Female residents believed that childbearing during training would have a negative impact on their careers—a phenomenon that has been previously well described [6, 25, 26]. A study of female orthopaedic residents in the United States found that 60% of residents experienced bias about women having children during residency [6]. In a survey of Canadian female otolaryngologists-head and neck surgeons, significantly more women than men stated that maternity leave impacted advancement opportunities (32% vs. 7%) and salary/remuneration (71% vs. 24%) (p < 0.001) [16]. Similarly, in our study, we found that women were much more likely to feel negative stigma associated with pregnancy during training (62.7% vs 29.3%, p = 0.01). Conversely, men were less likely to report hearing negative comments by staff or colleagues about pregnancy during training (37.6% vs 54.5%, p<0.001). One respondent went on to say: “I was told on many occasions jokingly by attending staff not to attempt getting pregnant in residency. I think that’s enough said.” One third of women and one fifth of men felt that pregnancy or parental leave during training had an impact on future job prospects.

Not only does pregnancy during training carry negative stigma, but it also affects pregnancy outcomes. A scoping review from 2023 found that infertility was frequently reported among female surgeons [18]. A systematic review published in 2020 found a wide range of reported obstetrical complication rates in surgical residents, ranging from 25–82%, compared to only 5–15% in the general US population [4]. Pregnant shift workers who work rotating shifts, longer hours (>40 hours/week) or night shifts are at increased risk of adverse pregnancy outcomes [10] as are residents and surgeons who work more than 60 hours/week [4, 27]. Despite our limited response rate, we found that almost two thirds of pregnant women in our study had a pregnancy complication, which is similar to reported rates in the literature for surgeons.

A study from Harvard Medical School on general surgeons who had a pregnancy during training found that 39% had strongly considered leaving surgical residency, and 29.5% would discourage female medical students from a surgical career because of the difficulties of balancing pregnancy and motherhood with surgical training [28]. The study authors suggested the challenges of having children during surgical residency may have significant workforce implications in the future [28]. To that end, one of our resident responders commented: “I feel my small residency program often struggles with… having (not) enough residents for the amount of work at each hospital site we cover. This leads to the feeling that taking time for pregnancy and paternal leave may be somewhat begrudgingly given, thought I believe it would be granted if I were to become pregnant.” In our study, 60% of residents felt that reduced work hours during pregnancy or parental leave had a negative impact on increasing the workload of others, with no difference between genders. “There is a lot of guilt that getting pregnant will mean that other residents need to pick up more call to cover the service.”, a resident stated. One way to support residents who become pregnant during training is to appropriately plan for the workforce implications of pregnancy and maternity leave to not unduly burden other residents. This may require the addition of physician extenders, night float rotations, or other unique strategies to manage having fewer residents covering overnight call.

Although surgical trainee parents still face significant difficulties, there have been improvements demonstrated over time. A study of Canadian plastic surgery residents, recent graduates, and staff found that of individuals who had children during residency, residents or recent graduates were more likely to have taken maternity or paternity leave compared to staff [29]. This suggests recent improvements in the culture of taking parental leave over time. A comparison of resident perceptions on pregnancy during training in the USA was performed based on survey responses in 2008 and 2015. They found that program directors and division chiefs were perceived to be more supportive of resident pregnancy in 2015 compared to 2008 [30]. One respondent in our study commented: “I am currently pregnant, and I am lucky to be in a residency program that is overall very supportive of parents, and it certainly has an influence on how well my pregnancy is going.” There must be greater institutional and collegial support for women to support their dual roles of both mothers and surgeons; with increased awareness, progress in policy and guideline development is under way [18, 31].

### Limitations

There are distinct limitations with our study. There was a low response rate of 11%; therefore, due to selection bias, our results should be considered a pilot study, and not a generalization of the entire Canadian surgical trainee population. Additionally, our results had a high proportion of orthopaedic, obstetric and gynecology, and general surgery residents. Thus, our results may not accurately reflect the perceptions of pregnancy and parenthood in all surgical subspecialities, especially specialities that may have a higher proportion of male or female surgical trainees and staff. There was a very high proportion of female respondents, and hence conclusions surrounding the male perspective on this topic may be limited. We neglected to ask respondents about their family structure and relationship status, and while we debate whether being uncoupled or not in a relationship would have skewed our results, we acknowledge this lack of demographic information. Our study is at risk of response bias, as residents who have stronger feelings about parenthood during training may be more likely to complete the survey. The wording of the questions might have also led to agreement bias in the responses. In our attempt to avoid recall bias by not asking male respondents about their female partners’ pregnancy complications, we did not intend to diminish the significance of such complications on the non-childbearing parent. Further study is required with buy-in from program directors and/or residency associations to encourage higher participation rates to get a better understanding of the entire training study population.

## Conclusion

The experiences of female trainees in Canada regarding pregnancy and parenthood during surgical training differ significantly from male trainees. Notably, female surgical trainees experience higher rates of pregnancy complications when compared to non-surgical counterparts, more negative stigma and bias, and other social and logistical challenges. This study highlights the need to create a culture in Canadian surgical training programs where both birthing and non-birthing parents are empowered and supported to take an active role in parenting to ultimately work towards achieving gender equity in surgical specialties.

## Supporting information

S1 File Parenthood in surgical residency survey. (PDF)
