# Supplementary material for: The double-edged scalpel: Experiences and perceptions of pregnancy and parenthood during Canadian surgical residency training
Source: PLoS One. 2024 Mar 27;19(3):e0301190. doi: 10.1371/journal.pone.0301190 (PMC10971744; doi:10.1371/journal.pone.0301190)
Supplement: S1 File — (PDF) [file pone.0301190.s001.pdf]

Parenthood in Surgical Residency

Start of Block: Default Question Block

Q1 **CONSENT: Parenthood During Surgical Training in Canada**

Who is conducting this study?

Principal Investigator:
Dr. Kelly Lefaivre, Vancouver General Hospital Orthopaedic Trauma, 604-875-5809
Kelly.lefaivre@vch.ca

Co-investigator:
Dr. Mikaela Peters PGY4, University of British Columbia Department of Orthopaedics, 604-362-5779
mikaelajpeters@alumni.ubc.ca

Who is sponsoring this study?

This study is being funded through a grant from Resident Doctors of BC.

Why are we doing this study?

You are being invited to take part in this research study because you are a surgery resident or fellow enrolled in a Canadian program. We want to learn more about perceptions and experiences of parenthood during surgical training.

What happens if I agree to participate?

This study involves a 10-15 minute survey about your perceptions of parenthood during surgical training. If you became a parent during surgical training, we will also ask about your children and your experiences. If the questionnaire is completed, it will be assumed that consent has been given.

The results of this study may be presented at conferences or published in academic journal articles. If you would like a copy of any future publications, there will be an opportunity on the online questionnaire for you to provide your email address. This email address will not be linked to any of the questions you answered.

Are there any risks to this study?

We do not think there is anything in this study that could harm you. Some of the questions may be upsetting or triggering. Please contact your local Residency Wellness program or the study staff if you have any concerns.

Are there any benefits to this study?

We do not think taking part in this study will help you. However, in the future, the results of this study may be used to improve the experiences of parents during surgical training.

How will my identity be protected?

Personal information, such as the email address provided for future published works, will not be linked to the rest of the questionnaire. Your confidentiality will be respected.

Survey results may be required to be “open access” for journal article submission. This means that other researchers may have access to pooled survey results. No identifying information will be linked to your survey results.

The survey company Qualtrics will be used to collect your answers to an online questionnaire. All data will be encrypted and hosted in Canada.

Will you be paid for your time?

Unfortunately you will not receive any compensation for your time.

Who can you contact if you have questions about the study?

If you have any questions or concerns, please contact one of the study staff listed at the beginning of this document.

Who can you contact if you have complaints or concerns about this study?

If you have any concerns or complaints about your rights as a research participant and/or your experiences while participating in this study, contact the Research Participant Complaint Line in the UBC Office of Research Ethics at 604-822-8598 or if long distance email RSIL@ors.ubc.ca or call toll free 1-877-822-8598.

Participant consent

Consent is assumed if you proceed to the questionnaire. You may choose to pull out of the study prior to submission by closing your internet browser. You will not be able to withdraw after the survey has been submitted.

- [ ] I consent (1)
- [ ] I do not consent and wish to end the survey (2)

Skip To: End of Survey If CONSENT: Parenthood During Surgical Training in CanadaWho is conducting this study?Principal Inve... = I do not consent and wish to end the survey

---

Q2 What is your age in years?

____

Q3 What gender do you identify as?

- ○ Male (1)
- ○ Female (2)
- ○ Non-binary / third gender (3)
- ○ Prefer not to say (4)

Supplement 1: Survey

Q4 What surgical specialty are you in?

- ○ Cardiac Surgery (1)
- ○ General Surgery (2)
- ○ Neurosurgery (3)
- ○ Obstetrics and Gynecology (4)
- ○ Orthopaedic Surgery (5)
- ○ Otolaryngology (6)
- ○ Plastic Surgery (7)
- ○ Urology (8)
- ○ Vascular Surgery (9)

Q5 What year of residency are you in?

- ○ PGY-1 (1)
- ○ PGY-2 (2)
- ○ PGY-3 (3)
- ○ PGY-4 (4)
- ○ PGY-5 (5)
- ○ PGY-6 (6)
- ○ PGY-7 (7)
- ○ PGY-8 (8)
- ○ Fellowship (9)

Q6 What province/ territory are you in?

- Alberta (1)
- British Columbia (2)
- Manitoba (3)
- New Brunswick (4)
- Newfoundland and Labrador (5)
- Northwest Territories (6)
- Nova Scotia (7)
- Nunavut (8)
- Ontario (9)
- Prince Edward Island (10)
- Quebec (11)
- Saskatchewan (12)
- Yukon (13)

Q7 How many children have you had during residency?

- ▢ 0 (1)
- ▢ 1 (2)
- ▢ 2 (3)
- ▢ 3 (4)
- ▢ 4+ (5)
- ▢ Currently pregnant (6)

Q45 Have you had a miscarriage during training? If yes, please elaborate.

- ○ Yes (4) ____
- ○ No (5)
- ○ Not applicable (6)

Q24 If applicable, what type of work does your partner do?

- ○ Stay at home caregiver (1)
- ○ Working part time or casual (2)
- ○ Working full time (3)
- ○ Not applicable (4)

End of Block: Default Question Block

Start of Block: Child 1

Q26 What stage of training was your first child born?

○ PGY-1 (1)

○ PGY-2 (2)

○ PGY-3 (3)

○ PGY-4 (4)

○ PGY-5 (5)

○ PGY-6 (6)

○ PGY-7 (7)

○ PGY-8 (8)

Page Break

Q27 Did you have any trouble conceiving your first child?

- Yes (1)
- No (2)

Q28 If you were pregnant, did you have any complications with your first child during pregnancy, delivery or postpartum?

- [ ] I was not pregnant with this child (1)
- [ ] Gestational diabetes (2)
- [ ] Intrauterine growth restriction (3)
- [ ] Preeclampsia (4)
- [ ] Eclampsia (5)
- [ ] Premature labour (6)
- [ ] Infections (UTI, GBS, influenza, COVID-19) (7)
- [ ] Stillbirth or neonatal loss (8)
- [ ] Low birth weight (9)
- [ ] Placenta previa (10)
- [ ] Low birth weight (11)
- [ ] Postpartum anxiety or depression (12)
- [ ] Pregnancy induced hypertension (13)
- [ ] Other (14) ____
- [ ] None (15)
- [ ] Click to write Choice 16 (17)

Q29 How long of a leave did you take with your first child? Please specify days, weeks, or months

____

Q30 Rate your satisfaction with the amount and quality of leave you were able to take with your first child

- ○ Very unsatisfied (1)
- ○ Unsatisfied (2)
- ○ Neutral (3)
- ○ Satisfied (4)
- ○ Very satisfied (5)

Q31 Were you able to work with reduced work hours during this pregnancy?

- ○ Yes (1)
- ○ No (2)
- ○ Somewhat (explain) (3) ____
- ○ Not applicable, I was not pregnant (4)

Q32 Were you able to work with reduced work hours after you returned to work, or if you did not take a leave, after your first child was born?

- ○ Yes (1)
- ○ No (2)
- ○ Somewhat (explain) (3) ____

Q33 Have you experienced stigma or bias due to your status as a parent? If yes, please explain

- ○ Yes (1) ____
- ○ Maybe (2) ____
- ○ No (3)

End of Block: Child 1

Start of Block: Child 2

Q34 What stage of training was your second child born?

- ○ PGY-1 (1)
- ○ PGY-2 (2)
- ○ PGY-3 (3)
- ○ PGY-4 (4)
- ○ PGY-5 (5)
- ○ PGY-6 (6)
- ○ PGY-7 (7)
- ○ PGY-8 (8)

Q35 Did you have any trouble conceiving your second child?

- ○ Yes (1)
- ○ No (2)

Q36 If you were pregnant, did you have any complications with your second child during pregnancy, delivery or postpartum?

- ☐ I was not pregnant with this child (1)
- ☐ Gestational diabetes (2)
- ☐ Intrauterine growth restriction (3)
- ☐ Preeclampsia (4)
- ☐ Eclampsia (5)
- ☐ Premature labour (6)
- ☐ Infections (UTI, GBS, influenza, COVID-19) (7)
- ☐ Stillbirth or neonatal loss (8)
- ☐ Low birth weight (9)
- ☐ Placenta previa (10)
- ☐ Low birth weight (11)
- ☐ Postpartum anxiety or depression (12)
- ☐ Pregnancy induced hypertension (13)
- ☐ Other (14) ____
- ☐ None (15)

Q37 How long of a leave did you take with your second child? Please specify days, weeks, or months

____

Q38 Rate your satisfaction with the amount and quality of leave you were able to take with your second child

- ◯ Very unsatisfied (1)
- ◯ Unsatisfied (2)
- ◯ Neutral (3)
- ◯ Satisfied (4)
- ◯ Very satisfied (5)

Q39 Were you able to work with reduced work hours during this pregnancy?

- ◯ Yes (1)
- ◯ No (2)
- ◯ Somewhat (explain) (3) ____
- ◯ Not applicable, I was not pregnant (4)

Q40 Were you able to work with reduced work hours after you returned to work, or if you did not take a leave, after your second child was born?

○ Yes (1)

○ No (2)

○ Somewhat (explain) (3) ____

End of Block: Child 2

Start of Block: Child 3

Q42 What stage of training was your third child born?

○ PGY-1 (1)

○ PGY-2 (2)

○ PGY-3 (3)

○ PGY-4 (4)

○ PGY-5 (5)

○ PGY-6 (6)

○ PGY-7 (7)

○ PGY-8 (8)

Q43 Did you have any trouble conceiving your third child?

○ Yes (1)

○ No (2)

Q44 If you were pregnant, did you have any complications with your third child in pregnancy, delivery or postpartum?

- ☐ I was not pregnant with this child (1)
- ☐ Gestational diabetes (2)
- ☐ Intrauterine growth restriction (3)
- ☐ Preeclampsia (4)
- ☐ Eclampsia (5)
- ☐ Premature labour (6)
- ☐ Infections (UTI, GBS, influenza, COVID-19) (7)
- ☐ Stillbirth or neonatal loss (8)
- ☐ Low birth weight (9)
- ☐ Placenta previa (10)
- ☐ Low birth weight (11)
- ☐ Postpartum anxiety or depression (12)
- ☐ Pregnancy induced hypertension (13)
- ☐ Other (14) ____
- ☐ None (15)

Q45 How long of a leave did you take with your third child? Please specify days, weeks, or months

____

Q46 Rate your satisfaction with the amount and quality of leave you were able to take with your third child

- ○ Very unsatisfied (1)
- ○ Unsatisfied (2)
- ○ Neutral (3)
- ○ Satisfied (4)
- ○ Very satisfied (5)

Q47 Were you able to work with reduced work hours during this pregnancy?

- ○ Yes (1)
- ○ No (2)
- ○ Somewhat (explain) (3) ____
- ○ Not applicable, I was not pregnant (4)

Q48 Were you able to work with reduced work hours after you returned to work, or if you did not take a leave, after your third child was born?

- ◯ Yes (1) ____
- ◯ No (2)
- ◯ Somewhat (explain) (3) ____

End of Block: Child 3

Start of Block: Child 4

Q49 What stage of training was your fourth child born?

- ◯ PGY-1 (1)
- ◯ PGY-2 (2)
- ◯ PGY-3 (3)
- ◯ PGY-4 (4)
- ◯ PGY-5 (5)
- ◯ PGY-6 (6)
- ◯ PGY-7 (7)
- ◯ PGY-8 (8)

Q50 Did you have any trouble conceiving your fourth child?

- ◯ Yes (1)
- ◯ No (2)

Q51 If you were pregnant, did you have any complications in with your fourth child during pregnancy, delivery or postpartum?

- ☐ I was not pregnant with this child (1)
- ☐ Gestational diabetes (2)
- ☐ Intrauterine growth restriction (3)
- ☐ Preeclampsia (4)
- ☐ Eclampsia (5)
- ☐ Premature labour (6)
- ☐ Infections (UTI, GBS, influenza, COVID-19) (7)
- ☐ Stillbirth or neonatal loss (8)
- ☐ Low birth weight (9)
- ☐ Placenta previa (10)
- ☐ Low birth weight (11)
- ☐ Postpartum anxiety or depression (12)
- ☐ Pregnancy induced hypertension (13)
- ☐ Other (14) ____
- ☐ None (15)

Q52 How long of a leave did you take with your fourth child? Please specify days, weeks, or months

____

Q53 Rate your satisfaction with the amount and quality of leave you were able to take with your fourth child

- ○ Very unsatisfied (1)
- ○ Unsatisfied (2)
- ○ Neutral (3)
- ○ Satisfied (4)
- ○ Very satisfied (5)

Q54 Were you able to work with reduced work hours during this pregnancy?

- ○ Yes (1)
- ○ No (2)
- ○ Somewhat (explain) (3) ____
- ○ Not applicable, I was not pregnant (4)

Q55 Were you able to work with reduced work hours after you returned to work, or if you did not take a leave, after your fourth child was born?

- Yes (1) ____
- No (2)
- Somewhat (explain) (3) ____

End of Block: Child 4

Start of Block: Block 2

Q17 Did you delay becoming a parent due to residency?

- Yes (1)
- No (2)

Display This Question:

If Did you delay becoming a parent due to residency? = Yes

Q18 What was the reason for delaying becoming a parent? Select all that apply

- Personal preference (1)
- Wanted to avoid missing training time (2)
- Concern about lack of quality time to spend with family (3)
- To avoid negative perception by faculty and/or co-residents (4)
- Other: (5) ____

Display This Question:

If Did you delay becoming a parent due to residency? = Yes

Q19 How satisfied were you with the decision to delay becoming a parent?

- Extremely dissatisfied (1)
- Somewhat dissatisfied (2)
- Neither satisfied nor dissatisfied (3)
- Somewhat satisfied (4)
- Extremely satisfied (5)

Q20 Did you experience burnout, anxiety, or depression during training that required you to take a leave of absence in residency?

- Yes (1)
- No (2)
- Prefer not to answer (3)

Q21 Have you heard negative comments related to pregnancy during training from staff or co-residents?

- Yes (1)
- No (2)
- Unsure (3)

Q22 Would you have had a child or more children if you had more support from the residency program / residency program director?

- ○ Yes (1)
- ○ No (2)
- ○ Unsure (3)

End of Block: Block 2

Start of Block: Block 3

Q25 Please state your agreement with the following statements

	Strongly agree (1)	Agree (2)	Neither agree nor disagree (3)	Disagree (4)	Strongly disagree (5)
Reduced work hours during pregnancy and maternity leave negatively impact the workload of co-residents (1)	○	○	○	○	○
There is a negative stigma attached to being pregnant during training (2)	○	○	○	○	○
Pregnant residents should be offered modified duties including reduction/cessation of call, reduced standing, and other accommodations recommended by their care team (3)	○	○	○	○	○
Trainees should make up any call they miss during pregnancy or parental leave (4)	○	○	○	○	○
Residents should be offered maternity leave (5)	○	○	○	○	○
Residents should be offered paternity leave (6)	○	○	○	○	○
Surgeons exhibit an increased rate of complications during pregnancy compared to the general population (7)	○	○	○	○	○
Parental leave during training	○	○	○	○	○

affects competency at program completion (8)					
Parenthood during training affects competency at program completion (9)	○	○	○	○	○
Pregnancy during training has an impact on future job prospects (10)	○	○	○	○	○
Parental leave during training has an impact on future job prospects (11)	○	○	○	○	○
Parenthood during training has an impact on future job prospects (12)	○	○	○	○	○

End of Block: Block 3

Start of Block: Block 7

Q46 Is there anything else you'd like to share?

____

End of Block: Block 7
